# Characterisation of CorGlaes^®^ Pure 107 fibres for biomedical applications

**DOI:** 10.1007/s10856-016-5752-z

**Published:** 2016-08-31

**Authors:** Ross Colquhoun, Nikolaj Gadegaard, David M. Healy, K. Elizabeth Tanner

**Affiliations:** 1 Biomedical Engineering Division, School of Engineering, University of Glasgow, Glasgow, G12 8QQ UK; 2 Giltech Ltd, 12 North Harbour St, Ayr, KA8 8BN UK; 3 Present address: Scottish Enterprise, Glasgow, G2 8LU UK; 4 Present address: IDP Services Ltd., Ayr, KA7 4EG UK

## Abstract

A degradable ultraphosphate (55 mol % P_2_O_5_) quinternary phosphate glass composition has been characterised in terms of its chemical, mechanical and degradation properties both as a bulk material and after drawing into fibres. This glass formulation displayed a large processing window simplifying fibre drawing. The fibres displayed stiffness and strength of 65.5 ± 20.8 GPa and 426±143 MPa. While amorphous discs of the glass displayed a linear dissolution rate of 0.004 mg cm^−2^ h^−1^ at 37 °C, in a static solution with a reduction in media pH. Once drawn into fibres, the dissolution process dropped the pH to <2 in distilled water, phosphate buffer saline and corrected-simulated body fluid, displaying an autocatalytic effect with >90 % mass loss in 4 days, about seven times faster than anticipated for this solution rate. Only cell culture media was able to buffer the pH taking over a week for full fibre dissolution, however, still four times faster dissolution rate than as a bulk material. However, at early times the development of a HCA layer was seen indicating potential bioactivity. Thus, although initial analysis indicated potential orthopaedic implant applications, autocatalysis leads to accelerating degradation in vitro.

## 1 Introduction

Bone is a hierarchically structured bioceramic composite composed of an organic matrix reinforced by an inorganic ceramic phase. Furthermore, due to its dynamically responsive nature, bone can adapt according to the mechanical demands of its loading environment and continually repair itself through a naturally occurring remodelling process [1, 2]. However, high-energy trauma or bone resection can lead to delayed unions or non-unions. As a result, surgical intervention is required using fracture fixation devices and bone graft materials to stimulate fracture repair [3–5].

The use of conventional metallic fracture fixation devices can induce the stress shielding phenomena in the surrounding bone due to the large mismatch in elastic moduli between a metal implant and bone [6]. The absence of mechanical stimulus to the bone that can then lead to localised osteopenia, bone weakening and ultimately implant loosening or refracture of the bone. Furthermore, as they are permanent these devices may also require further surgery to remove them [7–10].

Phosphate glass fibres (PGFs) have gained attention for a range of biomedical applications including cell delivery vehicles, biosensors and for the regeneration of soft or hard tissues [11]. Additionally, PGFs have also gained interest as degradable reinforcing agents in resorbable polymer composite devices for bone fracture fixation plates, pins or screws [11–13]. This is due to their composition dependent properties (e.g., mechanical, physical and dissolution rate) that can be tailored through modifications to the type, number and concentration of modifier oxides in the composition. Being degradable with the potential to have mechanical properties close to those of cortical bone could also eliminate the need for secondary removal surgery and stress shielding [14]. Futhermore, the cationic species released during the dissolution of the fibres could also induce beneficial in vivo responses at the bony defect [15, 16].

CorGlaes^®^ (Giltech, Ayr, UK) is a range of soluble phosphate-based glasses that incorporate metal oxides into their composition to tailor the dissolution rates and active metal ion release profiles, with previous in vitro results demonstrating potential applications as an orthopaedic biomaterial [17]. Accordingly, this investigation has examined CorGlaes^®^ Pure 107, a phosphate glass, as a biomaterial and future degradable composite reinforcing agent by characterising the glass’s physical and chemical properties as well as its bioactivity.

## 2 Materials and methods

### 2.1 Materials

CorGlaes^®^ Pure 107 is a proprietary commercial ultraphosphate (55 mol% P_2_O_5_) quinternary composition glass developed by Giltech Ltd that substitutes specific concentrations (mol%) of magnesium (Mg) and zinc (Zn) oxides into the initial phosphate (P), calcium (Ca) and sodium (Na) oxides of the CorGlaes^®^ system. Melt quenched discs of 15 mm diameter by 2 mm thick were produced by pressing melted cullet inside a graphite mould. The discs were then annealed by being placed inside a 420 °C preheated oven, which was raised to 465 °C at 5 °C min^−1^ and held for 60 min prior to cooling to room temperature at 1 °C min^−1^.

CorGlaes^®^ Pure 107 fibres were manufactured using a gravity-fed melt spinning technique. A temperature controlled interchangeable ceramic clay bushing was positioned above a 0.637 m diameter (2 m circumference) stainless steel drum. Prior to fibre manufacturing, the drum was coated with Knittol^®^ fibre finish (Selco, India) to reduce fibre-to-fibre abrasion, thus reducing surface damage and providing protection from environmental moisture [18]. Approximately 350 g of cullet fragments were heated to 1000 °C at 1 °C min^−1^ and held for 4 h to produce a homogenous glass melt. The molten glass was then transferred to a clay bushing containing an array of 50 2 mm diameter holes preheated to 680–690 °C to produce a melt viscosity suitable for fibre manufacturing. Under gravity, glass droplets travelled down through the orifices and were subsequently drawn and collected around the drum rotating at 250 rpm. The aligned fibres were then removed, placed in polyethylene bags and stored inside a desiccator without annealing. Prior to testing, the fibres were washed in chloroform (VWR, USA) for 10 min in order to remove the Knittol^®^ lubricant, and then dried inside a fume cabinet for 15 min at ambient temperature.

### 2.2 Methods

#### 2.2.1 Fibre characterisation

The diameter of the fibre was determined from a 20 mm long isolated tow using a Sigma VP Scanning Electron Microscope (Carl Zeiss, Germany) according to Method C of BS ISO 11567:1995 [19]. Samples were prepared by sticking the fibres onto card using aluminium tape before sputter-coating with a thin layer of gold (Agar Scientific, UK). Cross-sectional images were captured in secondary electron and back scattered electron modes using 20 kV accelerating voltage and measurements were recorded using Zeiss SmartSEM software. The average cross-sectional area was calculated from the diameters of 30 fibres [19].

The CorGlaes^®^ Pure 107 glass fibre structure was characterised by X-Ray Diffraction (XRD) on crushed fibre samples using a D5000 X-ray diffractometer (Siemens AG, Germany) and monochromatic Cu K*α* radiation (1.5418 Å). The analysis was performed at a scanning step rate of 0.02° over 2θ values from 5° to 85° to assess the degree of crystallisation. FTIR spectra were recorded using a Spectrum One FTIR Spectrometer (PerkinElmer, USA) with an Attenuated Total Reflectance accessory. Scans were obtained in absorbance mode across the 4000–400 cm^−1^ wavenumber range at a resolution of 8 cm^−1^ with 16 scans performed per spectra and analysed using the equipment’s Spectrum^®^ software (PerkinElmer, USA). Raman spectroscopy was performed on 20 mm long fibres using a LabRAM HR (Horiba Jobin Yvon, France) with a Ventus 532 laser (Ventus, USA) for 1400–100 cm^−1^ wavenumbers. Spectra were collected in triplicate (*n* = 3) using a 532.17 nm laser line with a 10 % OD objective and 600 grating over a 10 s accumulation period.

Thermal analysis of the glass was evaluated using Differential Scanning Calorimetry (DSC) STA 449 F1 Jupiter^®^ – Simultaneous TGA-DSC equipment (Netzsch, Germany) on approximately 10 mm long fibres. The samples were heated to 1200 °C at 10 °C min^−1^ inside an inert nitrogen atmosphere before cooling at 30 °C min^−1^ using air as the purge gas. Phase transformation temperatures were analysed using Proteus^®^ software (Netzsch, Germany) and Eq. 1 used to determine the glass’s processing window (PW). The PW is considered to reflect a glass’s thermal stability (i.e. its tendency towards crystallisation) and due to its consistent values was selected over alternative models such as the Hruby criterion [20, 21].

1 $$PW = {T_{oc}} - {T_g}$$

where *PW *= processing window (°C), *T*_*oc*_ = glass crystallisation onset temperature (°C) and *T*_*g*_ = glass transition temperature (°C).

#### 2.2.2 Mechanical testing

Tensile testing of the fibres was conducted following ISO 11566:1996 using a Zwick/Roell Z2.0 (Zwick Roell, USA) tensile test machine with a 5N load cell at a crosshead speed of 1 mm min^−1^ [22]. Tensile test specimens were prepared by creating card mounting frames with a 25 mm gauge length. Individual fibres were isolated from an aligned fibre tow and fixed to the mounting frames using epoxy adhesive (Loctite, Germany). Frames were then secured using grips to the testing equipment before the sidewalls of the frame were cut to isolate the fibre. Force-displacement data was then collected for 50 fibre samples using Zwick/Roell TestXpert^®^ software.

To account for influences from the load train and gripping system of the tensile testing set up, a correction coefficient (also known as the system compliance factor) was determined by tensile testing fibres at 10, 20 and 30 mm gauge lengths. Five samples were tested for each gauge length and the force-displacement plots used to calculate Δ*L*/Δ*F* for each gauge length. The system compliance factor (*k*) was then determined by plotting Δ*L*/Δ*F* against gauge length and linear regression back to zero gauge length [22].

The strength of a glass or brittle ceramic material is generally dependent upon its fracture toughness (*K*_IC_) and critical flaw size (*a*). Weibull distribution statistics were used to characterise the statistical variation in fracture strength of a brittle material and are based on the ‘weakest link theory’, where the most significant flaw will control the overall strength of a material [23]. The Weibull distribution was used to examine the scatter of fracture strength from calculating the Weibull modulus (*m*). The tensile testing data was sorted by increasing tensile strength and the Weibull distribution plotted. The normalising stress (defined as the stress at which 63.2 % of the fibres had failed) was calculated [23].

#### 2.2.3 Dissolution studies

Surface modification of the CorGlaes^®^ Pure 107 fibres was performed using a 10 wt.% concentration of a 3-aminopropyl-triethoxy (APS) sizing agent following the protocol of Khan et al. [24]. A 1.5 g batch of fibres was immersed in 100 ml of 10 wt.% APS solution for 15 min inside a laminar fume hood before being washed with 50 ml of ethanol under vacuum filtration. The sizing agent was then cured by placing the treated fibres in an air circulated oven at 120 °C for 24 h.

Dissolution studies on the glass were performed on discs (*n* = 3) using distilled water (DW) at 0.18 cm^2^ ml^−1^ surface area-to-volume ratio (SA:V) and stored individually inside polystyrene universal tubes in a 37 °C incubator for 6 weeks. During immersion, samples were periodically removed, dried and weighed (±0.1 mg Pioneer^™^ analytical balance, Ohaus, USA) along with measurements of the dissolution media pH (HI 221 pH metre – Hanna Instruments, USA). These measurements were performed every 24 h over the first week and then weekly for 6 weeks, with the dissolution media replaced initially at each measurement point and then twice weekly. Wet and dry weights were used to calculate the weight loss, using Eq. 2, and plotted against the dissolution period (hours) to give the dissolution rate in mg cm^−2^ h^−1^ [16, 25, 26].

2 $$Weight\,loss\,per\,unit\,area = \frac{{{w_o} - {w_t}}}{A}$$

where *w*_*o*_ = initial sample weight (mg), *w*_t_ = sample weight at time *t*_*x*_ (mg) and *A* = initial sample surface area (cm^2^). Ca^2+^, Mg^2+^ and Zn^2+^ cation concentrations were assessed using an AAnaylst™ 400 Atomic Absorption Spectrometer (PerkinElmer, USA) at 422.7 nm (Ca^2+^), 285.2 nm (Mg^2+^) and 213.9 nm (Zn^2+^) wavelengths with a nitrous oxide-acetylene flame used to compensate for phosphate interference. Sodium (Na^+^) concentration was determined by flame emission using a 410 Flame Photometer (Sherwood Scientific, UK).

The weight loss and media pH during the dissolution of CorGlaes^®^ Pure 107 fibres was determined (*n* = 3) for 1, 2, 3, 4 and 7 days immersion. The dissolution studies were performed using DW, phosphate buffered saline (PBS), Dulbecco’s Modified Eagle’s Medium (DMEM) and corrected-simulated body fluid (c-SBF) along with a set of APS coated fibres tested in DW. PBS was produced by dissolving a PBS sachet (P5368-10PAK, Sigma-Aldrich, USA) in 1 l of DW. The c-SBF was prepared by sequentially adding each reagent to a polypropylene bottle containing 750 ml of DW. After the addition of the final chemical reagent, the solution pH was buffered to 7.25 at 37 °C using 1M HCl with further DW added to create a final solution volume of 1000 ml [27]. Corrected SBF accounts for the SO_4_^2−^ ion deficiency in the original SBF-K9 formulation [27–29]. Bacterial growth in DMEM (Sigma-Aldrich, UK) was inhibited by the addition of sodium azide (Sigma-Aldrich, USA) at a concentration of 0.1 vol% prior to fibre immersion. The initial ionic concentrations and ionic conductivity of the c-SBF, DMEM and PBS are listed in Table 1.

**Table 1:** Ionic concentrations of acellular DMEM cell culture media, c-SBF and PBS (Data from Kokubo et al. [28] and Lutišanová et al. [67])

Ionic concentration (mM)								
Media	Na^+^	K^−^	Ca^2+^	Mg^2+^	HCO_3_^−^	Cl^−^	HPO_4_^2−^	SO_4_^2−^
c-SBF	142.0	5.0	2.5	1.5	4.2	147.8	1.0	0.5
DMEM	154.6	5.37	1.82	0.8	44.0	120.5	1.0	0.8
PBS	350	5.19	–	–	–	233	10	–

Fibre dissolution samples used a 200 mg batch of 20 mm length fibres placed inside a 10 ml glass vial giving a 14.4 cm^2^ ml^−1^ SA:V ratio, with each sample in an individual glass vial placed in a 37 °C static incubator. The sample weights were recorded periodically and the media pH measured. At each dissolution period, the media was decanted and samples dried using an air circulated oven at 60 °C for 4 h. The dry weight loss was then determined. The media was not changed, with the exception of the DMEM that was replaced after 96 h in order to mimic the regular changes used during in vitro cell culture studies [29, 30].

The dissolution rate of the fibres normalised to the total calculated surface area of each batch was determined from the weight loss measurements after 24 h dissolution. To calculate the surface area in a 200 mg fibre batch, each fibre was assumed to be of uniform length (20 mm) and identical diameter. The total number of fibres per 200 mg batch and the subsequent total surface area (cm^2^) was then calculated from the theoretical weight of a single 20 mm length fibre using the known density of the glass (2.65 g cm^−3^) [30, 31].

#### 2.2.4 In vitro bioactivity assessment

The potential bioactivity of the CorGlaes^®^ Pure 107 PGFs was assessed using c-SBF [26, 27]. 26 mg batches of 20 mm length fibres were immersed in c-SBF at 1 cm^2^ ml^−1^ SA:V ratio inside polystyrene universal tubes at 37 °C. The media was not changed over the immersion period and samples were examined in triplicate (*n* = 3) for each incubation period of 0.5, 1, 3, 6, 12, 24, 48, 168 and 336 h (2 weeks) before being extracted and rinsed in acetone (VWR). Extracted fibres were then dried at 60 °C inside an air circulated oven for 3 hours before being stored in a desiccator prior to further analysis. The pH of the c-SBF was measured at each time point with the bioactivity of the fibres characterised by FTIR and Raman spectroscopy and scanning electron microscopy (SEM).

FTIR spectra were recorded in absorbance mode across the 4000–400 cm^−1^ wavenumber range at a resolution of 8 cm^−1^ with 16 scans performed per spectra. Raman spectroscopy was performed over the 1500–100 cm^−1^ wavenumber range in triplicate (*n* = 3) from a 532.17 nm laser line with a 10 % OD objective and 600 grating over a 10 s accumulation period. Images of the fibre surfaces were also captured using a Sigma VP SEM (Carl Zeiss, Germany) in Secondary Electron and Back Scattered Electron modes at 20 kV accelerating voltage.

## 3 Results

**Fig. 1:**
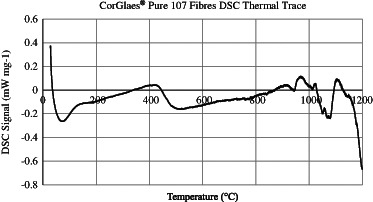
DSC thermogram of CorGlaes^®^ Pure 107 fibres over a thermal cycle from 25 °C to 1200 °C showing multiple crystallisation peaks and its liquidus temperature

**Fig. 2:**
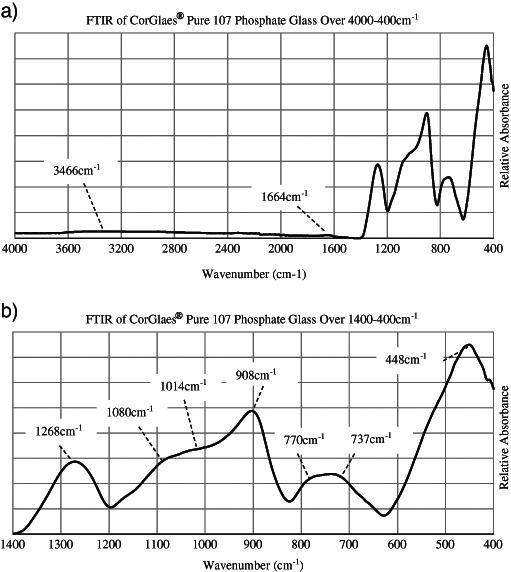
FTIR vibrational spectra of the CorGlaes^®^ Pure 107 phosphate glass over **a** the 4000–400 cm^−1^ and **b** the 1400–400 cm^−1^ wavenumber range showing absorption peaks and shoulders corresponding to the glass’s structure with the wavenumber of identified spectral features labelled

**Fig. 3:**
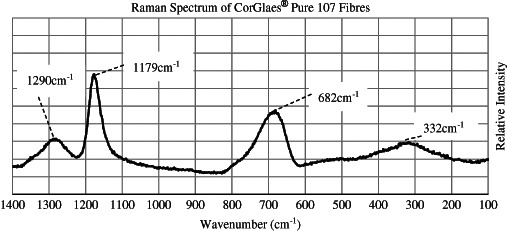
Raman spectrum of CorGlaes^®^ Pure 107 fibres over the 1400–100 cm^−1^ wavenumber range with peaks corresponding to the structural features of the CorGlaes^®^ Pure 107 composition

The XRD spectrum of the CorGlaes^®^ Pure 107 fibres showed no clearly defined crystallisation peaks, but a broad halo centred at 2θ ≈ 23°. The thermal properties obtained from DSC were determined from the intersection of tangents overlaid across the endothermic or exothermic events (Fig. 1) and are listed in Table 2 with the PW calculated using Eq. 1. FTIR traces over the complete mid-infrared spectrum (4000–400 cm^−1^) and 1400–400 cm^−1^ region are shown in Fig. 2. The position of these peaks, based on the literature, was assigned to the vibration of phosphate structural groups (Table 3) with the peaks at 3466 and 1664 cm^−1^ associated with the presence of moisture. The Raman spectrum obtained from the fibres is shown in Fig. 3 with the assignment of the spectral features to the phosphate structures listed in Table 4.

**Table 2:** Thermal properties of CorGlaes^®^ Pure 107 fibres

Thermal property	Temperature (°C)
Glass transition temperature (*T*_g_)	432
Crystallisation onset temperature (*T*_oc_)	775
Processing window (°C)	343
Crystallisation peak^1^ (*T*_p1_)	918
Crystallisation peak^2^ (*T*_p2_)	963
Crystallisation peak^3^ (*T*_p3_)	1022
Crystallisation peak^4^ (*T*_p4_)	1057
Liquidus temperature (*T*_lq_)	1074

**Table 3:** Assignments of spectral features identified from the CorGlaes^®^ Pure 107 FTIR spectrum over the 1400–400 cm^−1^ wavenumber range to the corresponding phosphate structural features

Feature	Wavenumber	Assignment
Peak	1268 cm^−1^	P = O [*v*_as_] associated with Q^3^ tetrahedra superposed with Q^2^ (PO_2_)^−^ [*v*_*as*_]
Peak/shoulder	1080 cm^−1^	Q^2^ (PO_2_)^−^ symmetric stretching [*v*_*s*_]
Peak	1014 cm^−1^	(P–O–P) asymmetric stretching [*v*_*as*_] (small metaphosphate rings)
Peak	908 cm^−1^	(P–O–P) asymmetric stretching [*v*_*as*_] (chains)
Peak	770/737 cm^−1^	(P–O–P) symmetric stretching [*v*_*s*_] (rings)
Peak	448 cm^−1^	Bending vibrations of bridging phosphorous *δ*(O–P–O) and/or *δ*(P=O)

**Table 4:** Assignments of spectral features identified in the Raman spectrum of the CorGlaes^®^ Pure 107 fibres to the phosphate structural features

Feature	Wavenumber	Assignment
Peak	1290 cm^−1^	(PO_2_) asymmetric [*v*_*as*_]
Peak	1179 cm^−1^	(PO_2_) symmetric [*v*_*s*_]
Peak	682 cm^−1^	(P–O–P) symmetric [*v*_*s*_]
Peak	332 cm^−1^	(P–O–P) bending vibration mode

**Fig. 4:**
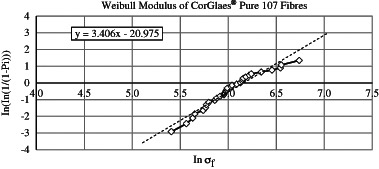
Determination of Weibull modulus (*m*) from CorGlaes^®^ Pure 107 fibre tensile test data based on the gradient of the plotted trendline

The average diameter of the fibres was 20.85 ± 3.25 μm with a range of 16.07–27.06 µm, giving an average cross-sectional area (*A*_f_) of 349.14 µm^2^. The mechanical testing gave an average tensile strength and tensile modulus of 426 ± 143 MPa and 65.5 ± 20.8 GPa, respectively and a Weibull modulus (*m*) of 3.406 with a normalising stress of 525 MPa (*σ*_0_) (Fig. 4).

**Fig. 5:**
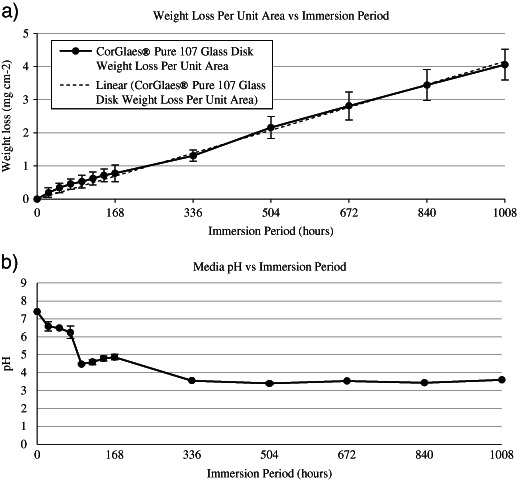
**a** Weight loss during dissolution of CorGlaes^®^ Pure 107 discs immersed in DW at 37 °C with the overlaid trendline plotted through the origin indicative of the glass’s dissolution rate, **b** extract pH of CorGlaes^®^ Pure 107 disc dissolution media showing the development of acidic pH after 72 h (standard deviation ≤ ±0.34)

**Fig. 6:**
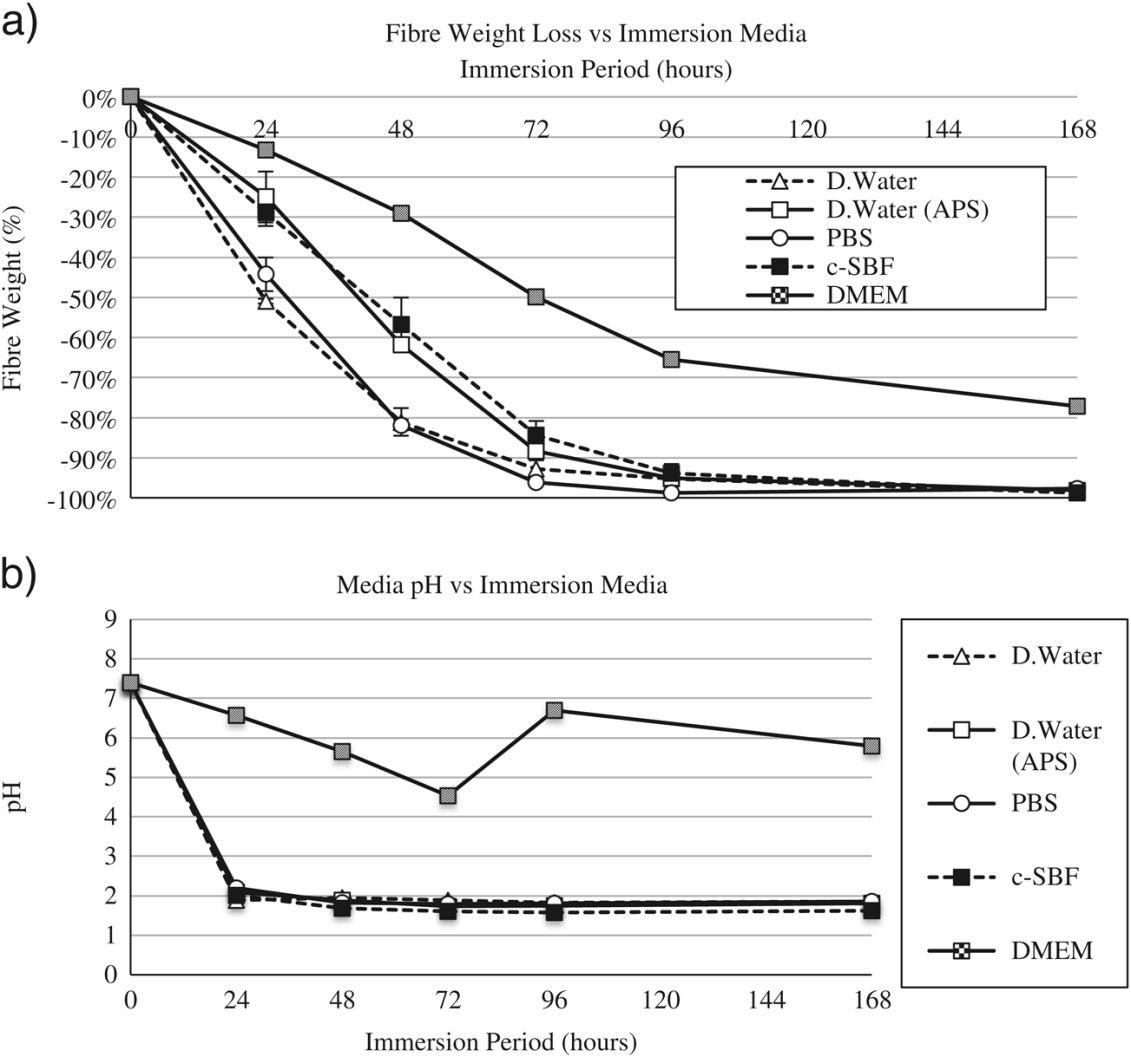
Comparison of CorGlaes^®^ Pure 107 fibre dissolution up to 168 h in various immersion media showing **a** the dry weight loss over time, **b** the media pH of the various immersion media upto 168 h with the replacement of DMEM at 96 h (standard deviation ≤ ± 0.07)

**Fig. 7:**
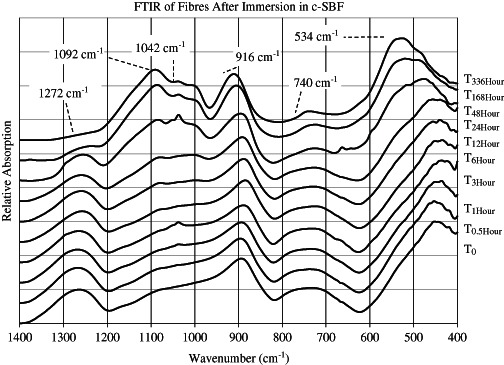
FTIR spectra from CorGlaes^®^ Pure 107 fibres after immersion in c-SBF for up to 336 h (2 weeks) with the formation and suppression of broad peaks over the immersion period labelled

**Fig. 8:**
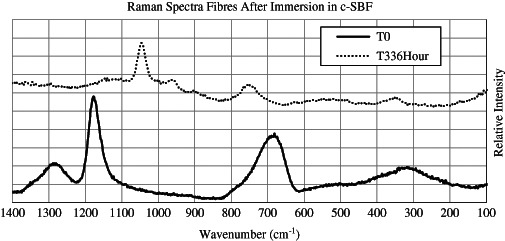
Comparison of Raman spectra obtained from CorGlaes^®^ Pure 107 fibres prior to bioactivity testing [T_0_] and after 336 h of immersion [T_336Hour_] in c-SBF at 37 °C

**Fig. 9:**
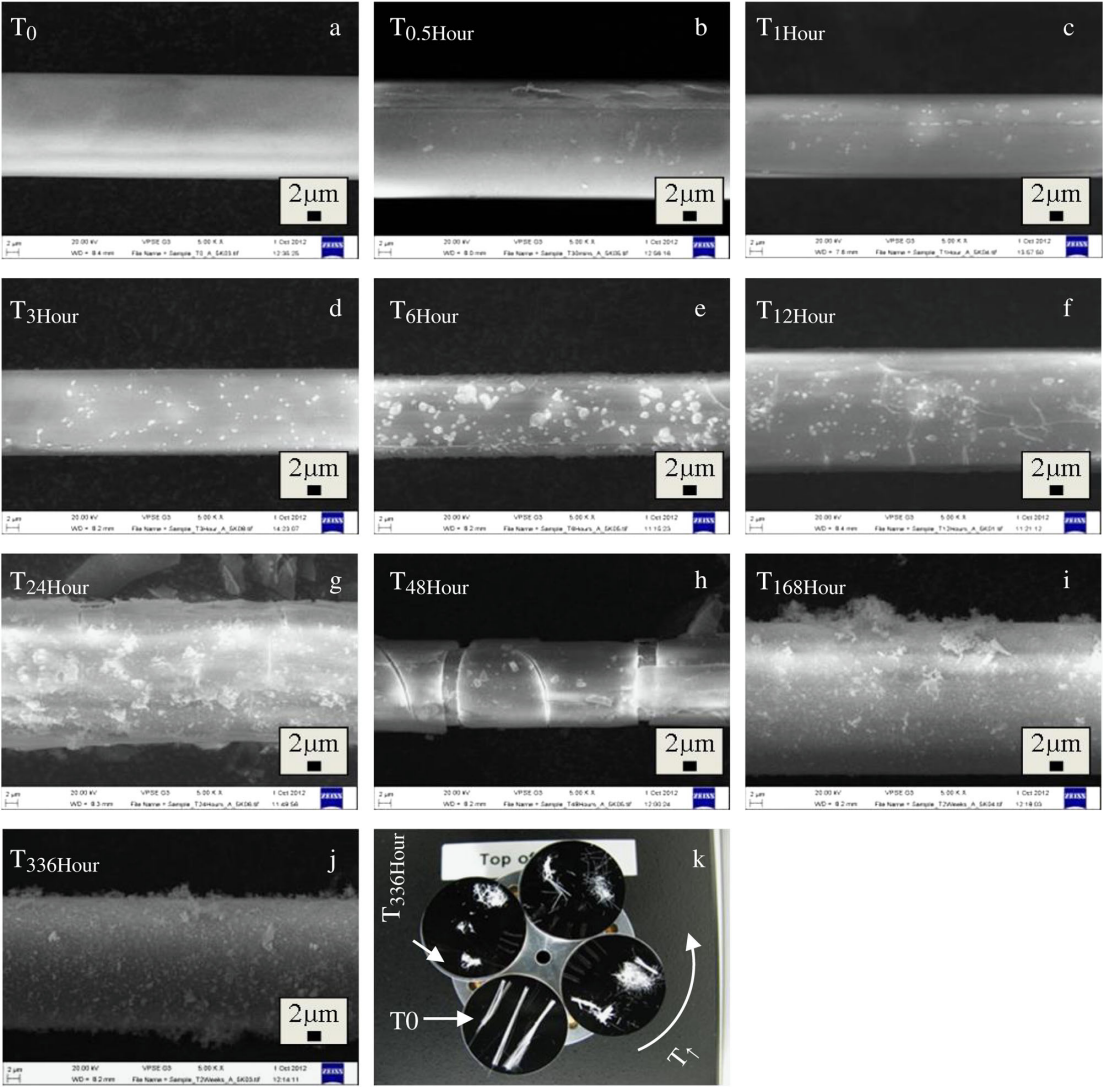
SEM Images of CorGlaes^®^ Pure 107 fibres prior to bioactivity testing [T_0_] and after immersion in c-SBF at various points over a 336 h period showing the changes across the fibre surface at **a** T_0_ **b** 0.5 **c** 1 **d** 3 **e** 6 **f** 12 **g** 24 **h** 48 **i** 168 **j** 336 h of immersion [all scale bars=2 µm] and **k** Image of CorGlaes^®^ Pure 107 fibres on 25 mm SEM mounting stubs after each immersion period in c-SBF prior to SEM imaging (moving counter-clockwise from T_0_ as indicated) showing the decreasing amount of retrievable fibre with increasing immersion time from T_0_→T_336Hour_

**Fig. 10:**
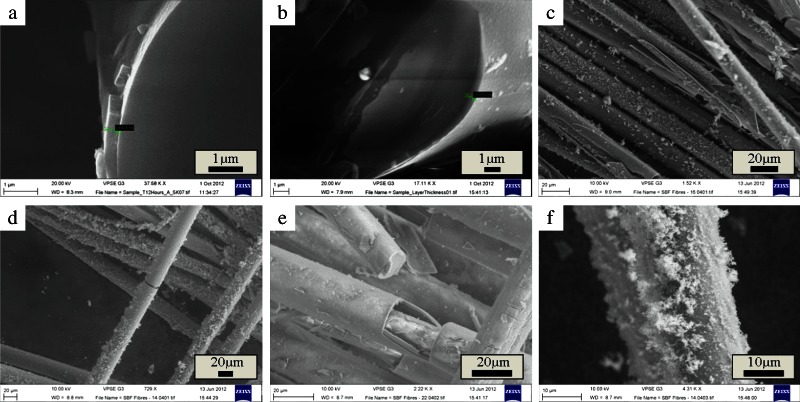
SEM images of CorGlaes^®^ Pure 107 fibres after immersion in c-SBF. **a**,**b** Fibre cross-section showing an outer surface layer of 400.4 nm and 550.6 nm thickness, respectively, after 12 h of immersion (scale bar=1 µm). **c**–**e** Surface precipitation and cracking on fibres after 168 h of immersion (scale bar=20 µm) and **f** surface deposition onto fibres after 336 h of immersion (scale bar=10 µm)

The weight loss per unit area and media pH over 1008 h (6 weeks) dissolution of the discs in DW are shown in Fig. 5. The dissolution rate was calculated as 0.004 mg cm^−2^ h^−1^. The dry weight loss and media pH over 24–168 h of fibre dissolution is shown in Fig. 6. The characterisation of fibre samples up to 336 h (2 weeks) immersion in c-SBF was assessed by FTIR (Fig. 7) and Raman (Fig. 8) with the corresponding SEM images of the fibres shown in Figs. 9, 10.

The fibres immersed in c-SBF changed from the initial FTIR and Raman spectra during immersion, with a whitish precipitate gradually forming on the fibres and at the bottom of the vials. This indicated the precipitation of compounds out of solution due to the super saturated conditions of the media and the ions released during glass fibre dissolution [36]. The handling of fibres also became increasingly difficult after 48 h due to increasing brittleness.

FTIR analysis of the fibres (Fig. 7) showed a shift in the broad absorption peaks initially centred at 893 cm^−1^ and ≈448 cm^−1^ [*T*_0_] to 916 and 534 cm^−1^, respectively [*T*_336Hour_], while a new broad peak spanning 1200–1000 cm^−1^ was also formed. This was coupled with partial and complete suppression of the peaks at 1268 and 737 cm^−1^, respectively [*T*_336Hour_]. A shift in the Raman spectra was also observed, but adequate results could only be collected from fibres after 336 h immersion due to fluorescence effects that produced poor signal-to-noise ratios. The Raman spectra (Fig. 8) after 336 h of immersion showed the suppression of previous [*T*_0_] peaks and the formation of broad peaks centred at 1042, 957, 750 and 356 cm^−1^. SEM analysis of the fibres revealed the simultaneous appearance of cracking and precipitate formation on the fibre surfaces as well as a change in the fibre morphology (Fig. 9). This appeared to follow a transition from a smooth pristine surface [*T*_0_] to one with surface cracking and peeling [*T*_6Hour_−*T*_48Hour_] that eventually returned to a smooth surface due to precipitate deposition. The immersion of fibres in c-SBF also saw a decrease in pH from 7.4 to ≈6.9 after 336 h immersion (Fig. 6b).

## 4 Discussion

The XRD profile of the CorGlaes^®^ Pure 107 fibres confirmed the amorphous structure and long-range disorder typical of glassy materials similar to the fibres investigated by Haque et al. [32]. This verified that no crystallisation had occurred during fibre manufacturing and that the glass feed temperatures (680–690 °C) were suitable for fibre production. The presence of a broad peak was in line with the literature and its location centred at 2θ≈23° was believed to correspond with the dominant crystalline phase that could be formed by this composition [33]. The position and shift in the location of an amorphous halo in an XRD trace is also considered as an indication of the degree of compactness in a glass’s network structure [34].

From the DSC thermogram (Fig. 1), CorGlaes^®^ Pure 107 possesses a larger PW than Bioglass^®^ 45S5 or other biomedical PGs due to its significantly higher *T*_oc_. This parameter is closely related to the viscosity of the glass melt and was considered to be a result of several contributing factors related to the composition, including its structural arrangement and the modifier oxides. Phosphate content has been reported by Ahmed et al. [35] to alter a glass’s thermal properties by acting on the structure and disrupting the phosphate network. Accordingly, ultraphosphate glasses are considered by Jones & Clare [20] to possess a greater thermal stability than metaphosphate and polyphosphate compositions. This is due to the structure of ultraphosphate glasses consisting of a 3D phosphate network with the combined Q^2^ phosphate chains and ring type structures further impeding the glass’s reorganisation into an arranged crystalline state by becoming entangled and increasing the melt viscosity [20]. The quinternary composition of CorGlaes^®^ Pure 107 probably supressed the onset of glass crystallisation by increasing the entropy of mixing and the subsequent energy barrier required for the formation of critical sized nuclei [20, 34, 35].

The inclusion of network modifier cations into the CorGlaes^®^ Pure 107 with sufficiently large charge-to-size ratios was also believed to influence the glass’s thermal stability. This is due to the expected strong ionic cross-linking in the phosphate network structure from the inclusion of Mg^2+^, Zn^2+^ and Ca^2+^ cations and their respective charge-to-size ratios (0.0232, 0.0227 and 0.0175). The network structure would then be more capable of suppressing the onset of crystallisation in line with previous results [20, 33, 36, 37]. However, previous studies have also shown that increased network stability is associated with a higher *T*_g_ due to the increased energy required to fracture the structural bonds that contradicts the comparatively low *T*_g_ found in these results. However, this could be accounted for by the general decrease in *T*_g_ found in ultraphosphate glasses compared to metaphosphate compositions as reported by Ahmed et al. [12, 20, 34, 37]. Based on previous metaphosphate glass compositions studied by Morikawa et al. [34], the large PW may also be attributed to a mixed cation effect between the Mg^2+^ and Ca^2+^ ions. This is believed to be related to the difference in polarity of the glass’s chemical bonds as a result of the higher electronegativity of magnesium than calcium [34].

The DSC thermogram (Fig. 1) also identified multiple crystallisation peaks (*T*_p1_, *T*_p2_, *T*_p3_, *T*_p4_) that were considered to represent the occurrence of surface and bulk crystallisation events as well the possibility of different crystalline phases. This was based on studies by Reynoso et al. [38] with broad (*T*_p1_, *T*_p2_) and sharp (*T*_p3_, *T*_p4_) crystallisation peaks corresponding to surface and bulk crystallisation events, respectively. The appearance of multiple crystallisation events is considered to be highly sensitive to particle size effects and consequently may not have appeared in the analysis of larger samples [38]. The larger surface area of fibres allows more surface nucleation and growth events to occur during crystallisation [38]. These results are in line with work reported by Jones & Clare [20], where a DSC thermogram of a 1 mm glass frit and fine glass powder of a P_2_O_5_–CaO–MgO–Na_2_O–TiO_2_ system showed the formation of two distinct crystalline phases in the powder form. These were later identified as calcium pyrophosphate (Ca_2_P_2_O_7_) and a mixed calcium magnesium pyrophosphate (CaMgP_2_O_7_) crystallisation phase. Given the relative similarity between the compositional components of this glass with the CorGlaes^®^ Pure 107 composition it would be anticipated that the crystalline phases may be similar [20, 38].

The occurrence of bulk crystalline phases (*T*_p3_, *T*_p4_) at temperatures above the 1000 °C melt temperature was probably due to the decrease in melt viscosity. As discussed by Jones & Clare [20], the tendency of a glass to crystallise is dependent upon its melt viscosity thus the higher temperature would have allowed the formation of crystalline phases due to the increased molecular mobility of the phosphate chains allowing reorganisation into a crystalline state. The identification of the liquidus temperature at 1074 °C indicated that a higher melt temperature (>1074 °C) should be considered for melt quenched applications for this glass. However, given that no crystallisation was found in the XRD results of the fibres there is also sufficient counter evidence to support the current manufacturing methods [20, 39].

The FTIR spectrum (Fig. 2) is believed to be a convolution of various overlapping Gaussian components that prevented the complete identification of all the phosphate structural groups. This band overlapping was likely due to the glass’s structural disorder from the presence of long P–O–P chains and rings in the Q^2^ structural units that would permit additional vibrational modes and account for the generally broad nature of the absorption peaks [40]. Furthermore, the assignment of wavenumber peaks to specific phosphate groups (i.e., Q^3^, Q^2^, Q^1^, Q^0^ units) is open to interpretation given the relative shifts in absorbance peaks produced by different glass compositions, is due to changes in the structural features and bond strengths within the glass’s network from the effects of its modifier oxides. Consequently, the phosphate structural groups are typically categorised over a range of wavenumbers [40].

From the results shown in Fig. 2 the assignment of the absorption peaks to Q^3^ and Q^2^ phosphate groups (Table 3) based on the available literature appeared to confirm the ultraphosphate composition of the CorGlaes^®^ Pure 107 glass. These assignments were also found to be in agreement with the generalised ranges described by Moustafa & El-Egili [40] due to the vibration of Q^2^ P–O–P chain and ring type structures at 737 cm^−1^, 770 cm^−1^, 908 cm^−1^ and 1014 cm^−1^. However, the assignment of bands at 1014 cm^−1^ is challenged by its association with Q^3^ P–O [*v*_*as*_] and thus despite agreeing with the consensus of data, some uncertainty remains in the current assignments [42]. Further identification of the structural phosphate groups within the composition could be achieved through deconvolution of the FTIR spectrum to resolve the broad peaks [40–42]. The broad but low intensity absorption peaks centred at ≈3466 cm^−1^ and ≈1666 cm^−1^ (Fig. 2) were assigned to atmospheric moisture absorbed on the glass surface [40, 42, 43]. Along with the DSC and XRD, Raman spectroscopy of the fibres identified Q^3^ and Q^2^ phosphate structural units that coordinated with its ultraphosphate composition (55 mol% P_2_O_5_) agreeing with previous FTIR results [44].

The 20.85 ± 3.25 µm diameter of the CorGlaes^®^ Pure 107 PGFs was in line with 10–20 µm diameters typically used in fibre reinforced composites and in line with those used in the development of similar degradable PGF reinforced composites [45–47]. The high aspect ratios of continuous fibres, with the large surface area per unit volume, allows good stress transfer and reinforcement throughout a composite material. The results consequently indicated that the current manufacturing specifications were highly suitable for the production of fibres as a composite reinforcing phase [45–47].

The stress-strain plots were characteristic of a brittle material, showing no significant plastic deformation prior to fracture. However, from the 50 samples prepared only 32 results were used for further data analysis. This was due to the premature failure of fibre samples during testing, or the sporadic occurrence of multiple fractures in a single sample that was evident by the multiple deviations in the force-displacement plot, this was attributed to human error during sample preparation leading to two or more fibres being mounted in one sample. Data was also excluded where fracture had occurred close to the adhesive.

**Fig. 11:**
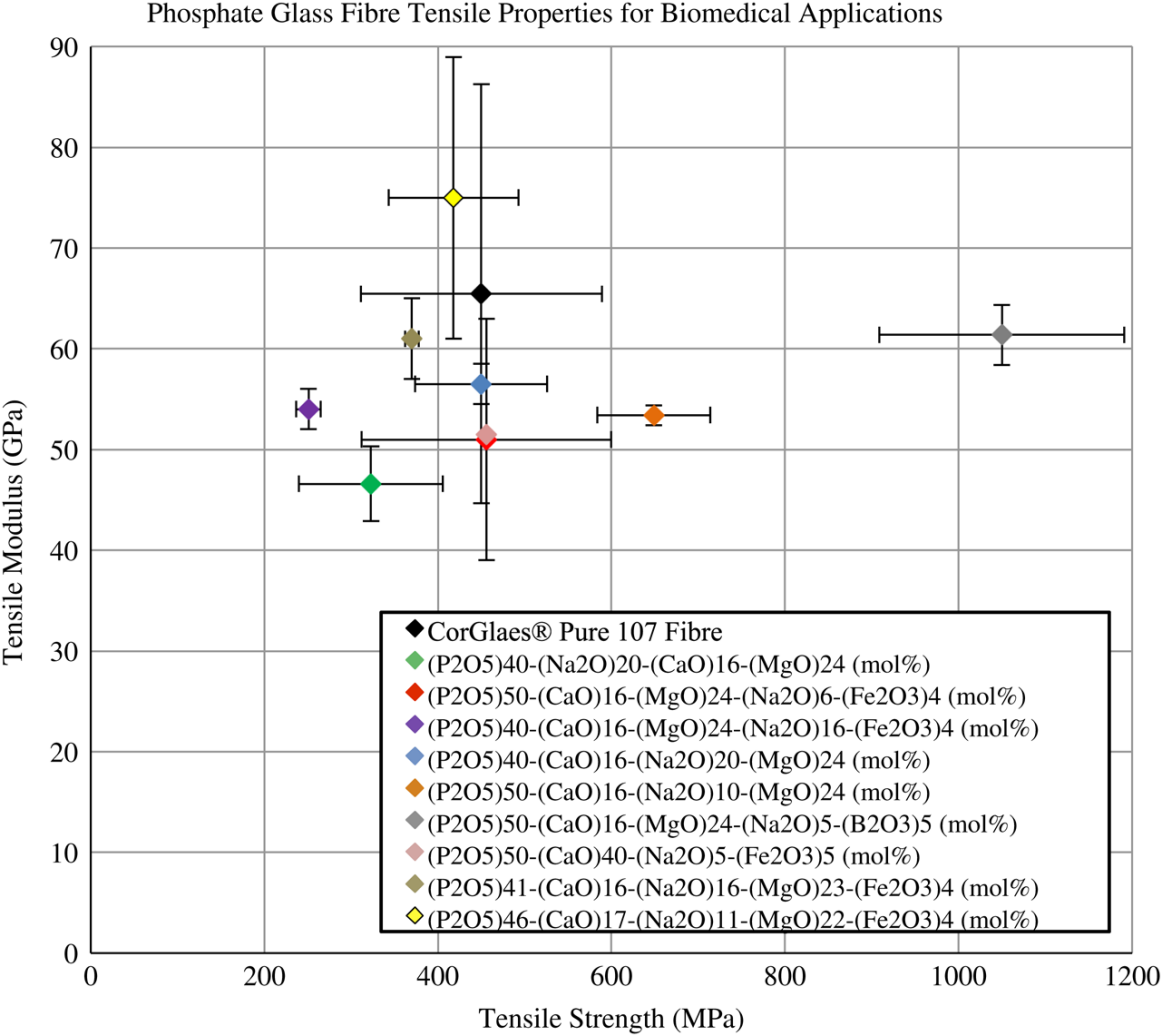
Comparison of CorGlaes^®^ Pure 107 fibre tensile properties with alternate phosphate glass fibre compositions of similar ≈20 µm diameter, tested according to ISO 11566:1996 [23, 30–32, 48, 62]

Compared to the bulk glass, the tensile modulus of the fibres was significantly higher. The fibre drawing process preferentially selects the strong P–O–P bonds and generates a degree of molecular chain alignment along the fibres long axis. Such chain alignment contradicts the glass’s natural isotropic structure (as found in glass monoliths) to produce fibres with anisotropic optical and mechanical properties [25, 48]. The average tensile modulus was also found to be in the upper range when compared with other biomedical PGF compositions (Fig. 11).

The tensile modulus of a phosphate glass fibre is considered to be dependent upon the same factors as the bulk material (i.e., the network’s cationic field strength and atomic packing density) [48]. The tensile strength of phosphate glass is regulated by the cross-link density and chain lengths of the phosphate backbone. The tensile strength of the CorGlaes^®^ Pure 107 is expected to benefit from the presence of magnesium and zinc which can potentially act as intermediate oxides to increase the glass’s cross-link density, or chain length by being incorporated into the P–O–P backbone. Yet the large standard deviation in the tensile strength and tensile modulus of the fibres has also made comparisons with alternate glass fibres difficult. This is attributed to factors related to both fibre manufacturing and test sample preparation methods [48]. Due to constrictions in the testing technique and limited fibre samples successfully recovered after fracture, an accurate correlation between the tensile test results and the individual sample fibre diameter was not possible. Thus the averaged fibre diameter of 20.85 µm was used, ignoring variations in the diameter. Variations would have arisen during fibre manufacturing due to changes in the hydrostatic pressure in the glass melt, and thus mass flow through the bushing nozzles, as the melt volume reduced [49]. Consequently, fibre diameters obtained at the start of manufacturing were likely to be larger than those at the end.

The large variation in the tensile strengths of these fibres was expected given the similar range reported in previous results (Fig. 11). These were attributed to the introduction of surface defects in the fibres that were probably generated during the manufacturing of the tensile testing samples (i.e., cutting and handling of the fibres). Yet the presence of such flaws are considered to be an accurate representation of the practical fibre properties due to the handling of fibres experienced during composite manufacturing [50]. This is in line with comments by Haque et al. who discussed the large number of studies dedicated to the ‘practical strength’ of glasses due to the influence of surface quality on their mechanical performance [32].

**Fig. 12:**
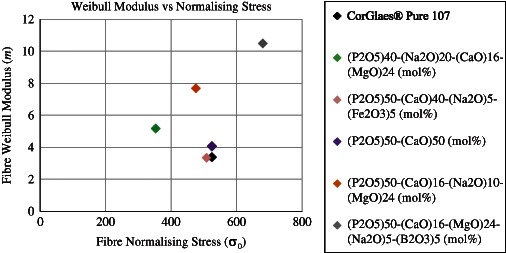
Comparison of CorGlaes^®^ Pure 107 fibre Weibull modulus (*m*) and normalising stress (*σ*_o_) with alternate PGFs tested according to ISO 11566:1996 [23, 30, 31, 48, 51]

The observed variation in tensile strength was accounted for by a Weibull distribution with the Weibull modulus (*m*) providing a dimensionless measure of the strength distribution throughout the fibres. The CorGlaes^®^ Pure 107 fibres gave a Weibull modulus of *m* = 3.41 with a normalising stress (*σ*_0_) of 525 MPa. This Weibull modulus value falls within the range reported for ceramic materials (*m* = 2–15), but is significantly lower than those typically found in commercial grade glass fibres (*m* = 10–30). A Weibull modulus below 4 also indicates that the flaws present on the fibres are not evenly distributed throughout the material and suggest some continuity. The Weibull modulus was also found to be comparable to values reported during the tensile testing of (P_2_O_5_)_50_–(CaO)_40_–(Na_2_O)_5_–(Fe_2_O_3_)_5_ (mol%) fibres (*m* = 3.37) using the same testing method [23]. Other studies by have demonstrated the ability to produce PGFs with Weibull moduli (*m*) as high as 7.7–10.5 (Fig. 12) [48, 51, 52].

Immersion of the CorGlaes^®^ Pure 107 discs in DW at 37 °C showed the dissolution rate to follow a two stage hydration and hydrolysis mechanism [53]. This was shown by the gradually decreasing dissolution rate over the first 168 h (1 week) of immersion followed by a more linear profile. From the plotted trendline through the origin, a dissolution rate (D_r_) was determined to be 4 × 10^−3^ mg cm^−2^ hr^−1^. From previous in vitro studies using macrophages, this rate was within the limits deemed acceptable for cell anchorage (1.7 × 10^−2^ mg cm^−2^ h^−1^), but too rapid for cell proliferation (3 × 10^−3^ mg cm^−2^ hr^−1^) [54]. However, a direct comparison is obscured by the high SA:V ratio used in the testing procedure of these fibres (14.4 cm^2^ ml^−1^) compared to most alternate methods (≈7.3 cm^2^ ml^−1^). The SA:V ratio will have a significant effect on fibre dissolution rate and would make the autocatalytic effects more severe for the fibres [55]. However, given that high SA:V ratios would be encountered at the fibre-matrix interface in a composite material during its degradation, the dissolution rates and pH values measured here were considered to provide a better prediction of the fibre behaviour as composite reinforcement. The use of annealing treatments has also been shown to reduce the dissolution rate of PGFs compared to their ‘as-made’ state and may be of interest for further processing of these fibres [31, 32, 48, 55, 56]. Furthermore, the ionic concentrations during sample immersion also showed the expected release of calcium, sodium, magnesium and zinc cations [53, 57].

The initial 24 h saw the immersion media pH fall to ≈6.5 due to the formation of carbonic acid due to carbon dioxide being dissolved from the atmosphere into the media. However, such pH values (i.e., 5.8 < pH < 6.5) are still considered relatively neutral compared to phosphate glass dissolution kinetics [57]. The further decrease in pH after 72 h (Fig. 5) was attributed to the release of various phosphate anions and their dissociation to form phosphoric acid (H_3_PO_4_) [23, 49, 58]. The initial release was believed to be from the annealed surface layer degrading across the disc samples due to moisture attacking the disc surface and cleaving the P–O–P chains produced in the glass during annealing [57]. The absence of any significant phosphate species being released into the media from dissolution of this outer surface layer may be due to the release of larger phosphate anionic species (i.e., P_2_O_7_^−4^, P_3_O_9_^−3^, P_3_O_10_^5−^) that were not included in the analysis [58, 59]. A significant increase in Na^+^ cation concentration after 96 h corresponded with a gradual increase in pH that was attributed to the consumption of H^+^ ions in the media from the hydration reactions during the glass dissolution [58–60].

The release of Na^+^ was also coupled with increases in Ca^2+^ and (PO_4_)^3−^ ion concentrations after 120 h of immersion from the saturation and hydrolysis of the glass’s phosphate network. Typically this is considered to signal the transition to a linear stage of glass dissolution and would appear to loosely correlate with the observed weight loss data (Fig. 5) after 144 to 168 h of immersion. Yet no significant increase in Zn^2+^ or Mg^2+^ release over the corresponding period was observed despite the relatively high concentration of magnesium in the sample composition compared to zinc. Accordingly, it was believed that the acidic environment generated by the sample dissolution (pH ≈4–4.8) led to the selective leaching of Zn^2+^ cations that has been shown to affect the release rate of Mg^2+^ [37].

The drop and subsequent plateau of the media pH to ≈3.5 after 336 h immersion was attributed to the reduced exchange and replacement of the dissolution media that allowed phosphate species to accumulate, leading to an increased concentration of phosphoric acid in the dissolution media in line with similar pH values recorded for other ultraphosphate glasses [25, 37]. Yet this decrease in pH failed to exhibit any significant autocatalysis effects and the linear sample dissolution rate continued (Fig. 5) [25, 37, 53, 61].

The dissolution of ‘as-made’ and surface treated fibres in different media (Fig. 6) showed distinct changes to the fibre dissolution rates over the initial 24 h. Continued immersion over the entire 168 h (1 week) period also revealed a non-linear weight loss profile after 72–96 h following an inverse exponential curve. Fibres immersed in DW (2.83 × 10^−2^ mg cm^−2^ h^−1^) and PBS (2.55 × 10^−2^ mg cm^−2^ h^−1^) displayed comparable dissolution rates while similar rates were observed between samples immersed in c-SBF (1.67 × 10^−2^ mg cm^−2^ hr^−1^) and silane treated fibres (1.44 × 10^−2^ mg cm^−2^ h^−1^) also over the initial 24 h. The dissolution of fibre samples in DW, c-SBF, PBS and silane treated fibres was also coupled with a drop in pH over 24 h plateauing at ≈2 (Fig. 6). This pH was significantly lower than that previously recorded from the dissolution of discs (Fig. 5) and was attributed to the increased SA:V ratio for the fibres (14.4 cm^2^ ml^−1^) compared to the bulk monolith samples (0.18 cm^2^ ml^−1^). Due to the static test conditions, this increased the accumulation of phosphate species and concentration of phosphoric acid in the media. In contrast, fibres immersed in DMEM showed the slowest rates of fibre dissolution (7.63 × 10^−3^ mg cm^−2^ h^−1^) that appeared to degrade in a linear fashion over a longer period compared to the other media. The pH also displayed a more gradual decrease reaching ≈4.5 at 72 h and corresponded with a visible change in the media colour from red to yellow before it was replaced with fresh DMEM after 96 h.

It is believed that PGFs will undergo the same dissolution mechanisms as their bulk monolith equivalent with the same compositional dependence. However, typically PGFs will typically degrade faster than the equivalent bulk sample [12, 25, 35]. This is due to the increased surface area and different mechanical/thermal histories between each form (e.g., the rapid air quenching of the fibres). The reduced fibre dissolution rates in DMEM were in line with observed trends [30], but remained below the preferred dissolution rate of fibres intended for composite reinforcement (2 × 10^−4^ mg cm^−2^ h^−1^) [62].

It has been previously reported that the ionic concentration of the immersion media can influence the rate of ionic diffusion from a phosphate glass (i.e., dissolution rate) [63]. Consequently it was believed that the initial ionic conductivity of each media would correspond with its ability to suppress the dissolution rate of the CorGlaes^®^ Pure 107 fibres. However, this trend was not strictly reflected in these results with similar dissolution rates observed in the DW (≈0 mS cm^−1^) and PBS (14.8 mS cm^−1^). This contrasted previous results where a reduced dissolution rate was observed when PGFs were immersed in PBS compared to DW [56]. However, the discrepancy between these two sets of results could be accounted for by the difference in glass composition and testing conditions. This study used a 10 ml static environment (SA:V = 14.4 cm^2^ ml^−1^) compared to Rinehart et al.’s 500 ml circulating flow system (SA:V = 0.15 cm^2^ ml^−1^) [56]. As a result the influence of the dissolution by-products on the glass dissolution process (specifically any autocatalysis effects) would have been substantially more severe in our study. The inhibitory effect of c-SBF on phosphate glass dissolution has been previously associated with the presence of Na^+^, Ca^2+^ and HPO_4_^2−^ ions [64]. However, c-SBF also contains Mg^2+^ and Ca^2+^ ions thus the reduced rates observed in c-SBF (19.53 mS cm^−1^) compared to PBS (14.8 mS cm^−1^) may be due to the absence of these ions in the PBS. Some of the visible precipitation within the c-SBF may have also influenced the weight loss measurements, However, no method of isolating the precipitated phases could be employed without potentially compromising the weight loss data [31, 64]. The decreasing rate of dissolution in the various media after 72–96 h was assumed to be due to the ionic saturation of the media and correlated with 80–85 % of the fibre weight being lost in all solutions except DMEM. This decrease was attributed to the saturation of the media with the least soluble ionic species [30, 31].

The improved durability of fibres degraded in DMEM media agreed with previous work and was believed to be due to the presence of a sodium bicarbonate (NaHCO_3_) pH buffering agent in the DMEM that retarded the decrease in media pH and autocatalysis effects during fibre dissolution [65]. This buffering agent subsequently accounted for the observed improvement in fibre durability compared to c-SBF despite their similar ionic composition (Fig. 6). The progressive change in DMEM colour was due to the presence of a phenol red pH indicator in the media that will change from its native red (at neutral) to yellow in acidic conditions. Silane surface treated fibres also showed a reduction in fibre dissolution rates that were comparable to that displayed by c-SBF. This was considered to indicate successful bonding between the sizing agent and fibre surface with the reduction in fibre dissolution rates stemming from the formation of a hydrophobic surface layer that retarded water diffusion into the glass [65–67].

Inspection of the FTIR spectra after the immersion of CorGlaes^®^ Pure 107 fibres in c-SBF found that these traces failed to correlate with the distinct wavenumber peaks associated with those of crystalline apatites [67]. The lack of any sharp, well-defined peaks in the FTIR spectra was indicative of an amorphous phase on the fibre surface and was believed to correspond with the precipitation of amorphous pyrophosphate salts [68, 69]. These included calcium pyrophosphate (Ca_2_P_2_O_7_), magnesium pyrophosphate (Mg_2_P_2_O_7_) and zinc pyrophosphate (Zn_2_P_2_O_7_) with the amorphous nature of these phases attributed to their rapid rate of precipitation from the super saturated c-SBF solution. Such compounds would correlate with the ionic constituents of the CorGlaes^®^ Pure 107 composition and have been previously reported to precipitate during PG dissolution [37, 63]. These phases are expected to form via hydrolysis mechanisms with the observed decrease in media pH over the immersion period resulting from the phosphoric acid (H_3_PO_4_) dissolution by-product [37, 63]. However, the precise identification of each amorphous phase from the available FTIR/Raman spectra is restricted due to the similar IR signals of these salts and associated band overlapping. Further identification could be conducted by post-annealing and repeated analysis via XRD and FTIR with deconvolution of the spectra to identify the crystalline phases [37, 63].

It has been reported that *β*-calcium pyrophosphate (*β*-CPP) can display bioactivity similar to that of hydroxyapatite (HA) and *β-*tricalcium phosphate (*β*-TCP). However, pyrophosphate ions (P_2_O_7_)^4−^ are also believed to supress HA crystallisation and have consequently been discussed as a method of influencing and controlling bone mineralisation [70–73]. Yet despite the potential of these pyrophosphate salts for in vivo applications, the inability of the CorGlaes^®^ Pure 107 glass fibres to form a more ‘traditional’ bioactive apatite layer (i.e., HCA) across the fibres surface during in vitro test conditions was expected [71–73]. Given that the fibre dissolution rate is considered to directly influence the resulting calcium phosphate phase, the precipitation of these salts was believed to reflect the high dissolution rate of the fibres [55]. Furthermore, the decrease in media pH produced from the glass fibre dissolution was believed to supress apatite formation despite the large quantities of calcium and phosphate released into the media. However, some PG compositions have demonstrated bioactivity in vivo despite failing to do so in vitro [43]. This could be due to the in vivo circulation of the extracellular fluid helping to maintain a physiological pH in the surrounding environment and allowing for apatite nucleation on the glass surface compared to the static environment encountered in vitro. Consequently the fibres may also be capable of displaying similar behaviour in vivo through improved pH regulation [36, 43, 72, 73]. However, it should also be considered that when applied as a composite reinforcing agent, the altered fibre dissolution rates as well as the large volume fractions of fibres may lead to the development of alternate phases when such composites are immersed in c-SBF.

The observed change in fibre morphology over the immersion period was attributed to the dissolution of the fibres and the subsequent cracking/peeling of an outer surface layer on the glass fibres. This change in morphology was in line with behaviour observed during PGF dissolution with additional SEM images of the fibres indicating a surface layer between 0.4–0.55 µm thick [48, 56].

## 5 Conclusions

Analysis of the CorGlaes^®^ Pure 107 glass found that this specific composition was capable of being manufactured into bulk monoliths that displayed dissolution rates suitable for cell adhesion. Yet the immersion of these samples also showed that acidic pH formed during sample dissolution may also be responsible for the selective ionic leaching (i.e., non-congruent dissolution) observed during sample dissolution.

This composition was also found suitable for producing fibres with mechanical properties comparable to those previously proposed for standalone cell transport vehicles or as degradable composite reinforcing agents. However, despite these mechanical properties, the immersion of ≈20 μm diameter fibre samples in DW found that this composition was susceptible to autocatalysis. Thus the dissolution rate of this fibre composition was deemed too rapid for application as a composite reinforcing agent. Further comparisons also showed that the choice of immersion media played a crucial role in retarding fibre dissolution rates due to their potential ability to regulate the media pH as well as create an ionic buffering effect. Under the current testing methods the fibres also failed to display the formation of a traditional bioactive HA layer when immersed in c-SBF. This was attributed to the rapid rate of fibre dissolution and reduction in pH that led to the formation of amorphous pyrophosphate salts.

The mechanical properties of the CorGlaes^®^ Pure 107 fibres appear to make them suitable as a potential composite reinforcing agent for investigating the initial mechanical properties of different novel composite configurations. However, the long-term performance of such composites would be limited without achieving a method of suitably controlling the media pH (and hence fibre dissolution rate) during the degradation of any potential composite samples [74].
